# Evidence Implicating CCNB1IP1, a RING Domain-Containing Protein Required for Meiotic Crossing Over in Mice, as an E3 SUMO Ligase

**DOI:** 10.3390/genes1030440

**Published:** 2010-12-02

**Authors:** Edward R. Strong, John C. Schimenti

**Affiliations:** Department of Biomedical Sciences and Center for Vertebrate Genomics, Cornell University, Ithaca, NY 14850, USA; E-Mail: ers37@cornell.edu

**Keywords:** meiosis, sumoylation, mouse, crossing over, recombination, yeast two hybrid

## Abstract

The RING domain-containing protein CCNB1IP1 (Cyclin B1 Interacting Protein 1) is a putative ubiquitin E3 ligase that is essential for chiasmata formation, and hence fertility, in mice. Previous studies in cultured cells indicated that CCNB1IP1 targets Cyclin B for degradation, thus playing a role in cell cycle regulation. Mice homozygous for a mutant allele (*mei4*) of *Ccnb1ip1* display no detectable phenotype other than meiotic failure from an absence of chiasmata. CCNB1IP1 is not conserved in key model organisms such as yeast and *Drosophila*, and there are no features of the protein that implicate clear mechanisms for a role in recombination. To gain insight into CCNB1IP1’s function in meiotic cells, we raised a specific antibody and determined that the protein appears in pachynema. This indicates that CCNB1IP1 is involved with crossover intermediate maturation, rather than early (leptotene) specification of a subset of SPO11-induced double strand breaks towards the crossover pathway. Additionally, a yeast 2-hybrid (Y2H) screen revealed that CCNB1IP1 interacts with SUMO2 and a set of proteins enriched for consensus sumoylation sites. The Y2H studies, combined with scrutiny of CCNB1IP1 domains, implicate this protein as an E3 ligase of the sumoylation cascade. We hypothesize CCNB1IP1 represents a novel meiosis-specific SUMO E3 ligase critical to resolution of recombination intermediates into mature chiasmata.

## 1. Introduction

In previous work, our lab conducted forward genetic mutagenesis screens to identify novel genes required for meiosis in mice [1,2]. One of the alleles induced by the point mutagen ENU (*N*-ethyl-*N*-nitrosourea), *mei4*, presented as a recessive male and female sterile. Histological and cytological analyses revealed abnormal alignment and distribution of chromosomes at metaphase/anaphase at the first meiotic division in spermatocytes and oocytes [3]. Immunocytological analyses revealed no abnormalities in non-crossover (NCO) recombination or chromosome synapsis through early pachynema. However, as the meiocytes entered diplonema, the homologous chromosomes failed to maintain interhomolog associations, suggesting an absence of chiasmata. This suspicion was confirmed by an absence of MLH1 and MLH3 foci on pachytene chromosomes [3]. The mismatch repair proteins are well established markers of chiasmata [4]. 

Positional cloning revealed that *mei4* is a mutant allele of *Ccnb1ip1* (also called *Hei10*), a gene not previously known to have a role in meiosis. *Ccnb1ip1* encodes a coiled-coil RING domain-containing protein, whereas *Ccnb1ip1^mei4^* bears a donor splice site mutation resulting in an aberrantly spliced transcript [3]. Studies of CCNB1IP1 in cultured somatic cells implicated a role for this putative ubiquitin E3 ligase in Cyclin B regulation, cell cycle progression, and cell invasion [5,6]. However, the exact function of CCNB1IP1 in meiotic recombination remains is unclear. A model proposed by Ward *et al.* posited that CCNB1IP1 disrupts association of CDK2 with CCNB3, possibly via ubiquitylation, thus permitting CDK2 to recruit or enable binding of MLH1 and MLH3 (and possibly other proteins) to designated crossover sites [3]. 

To better understand the role of CCNB1IP1 in recombination, and to gain possible support for the aforementioned model, we conducted a yeast two hybrid (Y2H) screen for interacting proteins in the mouse testis, characterized the temporal appearance of CCNB1IP1 during meiosis, and examined bioinformatically the domain structures of CCNB1IP1. Surprisingly, these studies implicate CCNB1IP1 as a SUMO (Small Ubiquitin-like Modifier) E3 ligase. SUMOylation modulates many behaviors of proteins, including interactions with other proteins, subcellular localization, and stabilization though competition with Ubiquitin for lysine residues [7]. The process of SUMO conjugation to target substrates is analogous to that of the well characterized Ub cascade; involving E1, E2 and E3 type ligases [8]. The role SUMO plays in meiosis remains largely unknown; however, immunolocalization studies in mammals have detected SUMO at sites of double strand breaks (DSBs) and at centromeric and heterochromatic regions, including the XY body of mouse pachytene spermatocytes [9,10,11,12]. Additionally, the singular SUMO E2 ligase, UBC9 (UBE2I in the mouse) localizes along synapsed chromosome cores during pachynema and diplonema [13,14]. The evidence we present in support of CCNB1IP1 as a potential SUMO E3 ligase has the potential to reveal hitherto unknown mechanisms in mammalian meiotic recombination.

## 2. Results and Discussion

### 2.1. Expression of CCNB1IP1 and CCNB1IP1^mei4^ During Spermatogenesis

CCNB1IP1 is essential for meiotic crossing-over in mice. In *S. cerevisiae*, although double Holliday junctions characteristic of crossover (CO) recombination appear in early-mid pachynema [15], the partitioning of DSBs to either the NCO or CO pathways is made much earlier, in late leptonema [16]. Like yeast, mammals have genetically distinct NCO and CO pathways [17]. Therefore, CCNB1IP1 may be required either for the specifying a subset of DSBs to the CO fate in leptonema, or subsequent processing of CO recombination intermediates in pachynema.

**Figure 1:**
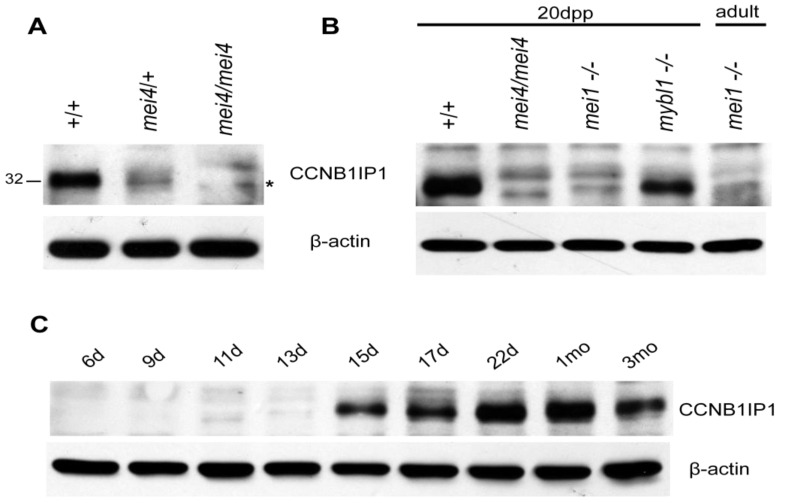
Western blot analysis of CCNB1IP1 expression in testis. (**A**) Polyclonal anti-CCNB1IP1 recognizes a ~32 kDa species in 20 dpp testis of WT and heterozygous *Ccnb1ip1^mei4^* animals (size in kDa is shown at left). This band is absent in mutants (third lane), but a lower band of ~30 kDa is evident that not present in WT (asterisk); (**B**) CCNB1IP1 is greatly decreased or absent from mutant testes that undergo meiotic arrest prior to pachynema (*Mei^-/-^*), but not those that progress to approximately diplonema (*Mybl1^-/-^*); (**C**) CCNB1IP1 in testis is first produced between days 13 and 15 dpp, coincident with onset of pachynema.

As a first step towards addressing this question, we generated an affinity-purified rabbit polyclonal antibody against *N*-terminal amino acids 1–245 of CCNB1IP1. The antibody recognized a protein slightly larger than 30 kDa (theoretical MW of CCNB1IP1 = 32 kDa) in Western blots of WT protein from 20 dpp (days post partum) and adult mouse testis. Juvenile *Ccnb1ip1^mei4^*/+ extracts had roughly half the amount of the 32 kDa species compared to WT animals. The 32 kDa species was completely lacking in homozygous mutants, consistent with it being CCNB1IP1 (Figure 1a). Notably, both heterozygous and homozygous testis extracts showed an additional, slightly smaller band on the Western blots (Figure 1a,b). This species was not as robust as wild-type CCNB1IP1, and it appeared to be more predominant in the homozygous mutants than in heterozygotes. Considering that the *Ccnb1ip1^mei4^* allele is predicted to encode a protein bearing an internal deletion of 24 amino acids (~2.7 kDa) [3], it is likely that the smaller species in the Western blot is this truncated protein. The mutant CCNB1IP1 allele may retain some function. However, the relatively lower amounts of the smaller species in both hetero- and homozygotes suggests that the CCNB1IP1*^mei4^* protein is less stable, more rapidly cleared, or translated at a lower efficiency than WT CCNB1IP1.

To further confirm the specificity of the antibody, we performed Western blot analysis of protein from 20 dpp testis extracted from several meiotic mutants (Figure 1b). The 32 kDa product is undetectable in *Ccnb1ip1^mei4^*^/*mei4*^ animals, but was present in mice homozygous for a mutant *Mybl1* allele that causes meiotic arrest at a stage of meiosis similar to that of *Ccnb1ip1^mei4^* spermatocytes (late pachynema/diplonema; [18]). This result indicates that the 32 kDa species is not a cross-reactive product from a class of cells that are missing in *Ccnb1ip1^mei4^*^/*mei4*^ testes. The product was present at low levels in *Mei1*/*Mei1* 20 dpp testis, in which meiosis arrests prior to entry into pachynema due to failed DSB formation and extensive asynapsis [19,20]. This suggests either that *Ccnb1ip1^mei4^* expression is either dependent upon DSB formation (which occurs in leptonema), or it initiates in pachytene spermatocytes.

To pinpoint the onset of CCNB1IP1 production, we took advantage of the coordinated first wave of spermatogenesis after birth. Leptotene, pachytene, late pachytene and diplotene cells appear *en masse* approximately 10, 14, 18, and >18 dpp, respectively [21,22]. As shown in Figure 1c, CCNB1IP1 appears between 13 and 15 dpp, spanning early-mid pachynema. CCNB1IP1 then persists throughout adulthood, although the data does not indicate if it is present in postmeiotic spermatids. These data indicate that CCNB1IP1 is not involved in partitioning DSBs to the CO pathway. Rather, expression after entry into pachynema suggests a requirement for processing CO recombination intermediates.

### 2.2. Identification of CCNB1IP1 Interacting Proteins

**Figure 2:**
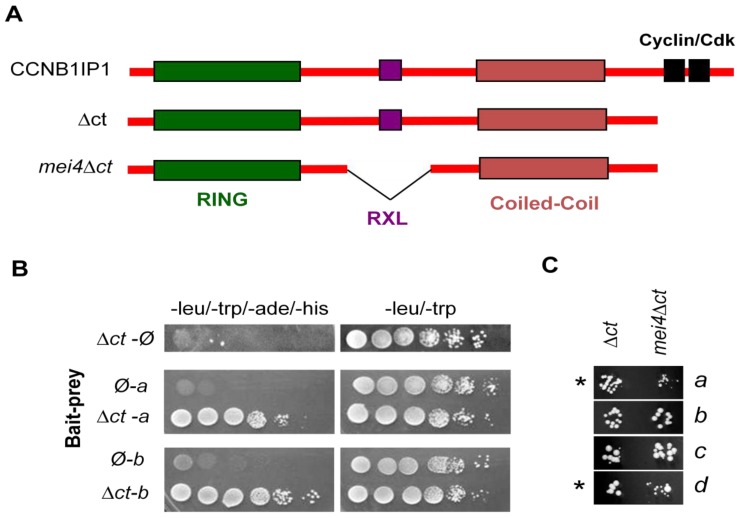
Yeast two-hybrid screen for CCNB1IP1 interacting proteins in the testis. (**A**) Structure of CCNB1IP1 and known motifs/domains. A *C*-terminal truncated construct was used as bait (∆*ct*). This lacks two potential phosphorylation sites of cyclin/cdk kinases. A bait construct with the deletion contained in the *Ccnb1ip1^mei4^* allele (*mei4*∆*ct*) is shown at the bottom; (**B**) Confirmation of prey clones under maximal selection stringency (ade and his) with leu and trp selection for presence of bait and prey plasmids; (**C**) Growth of yeast containing 4 different prey and either the ∆*ct* bait or the mei4∆*ct* bait. A subset of CCNB1IP1 interactors (asterisks) were found to reproducibly display weaker interaction with *mei4*∆*ct* than ∆*ct*, as assessed by vigor of individual colony growth. Ø = empty vector; a = EP400; b = OAZ3; c = 5730469M10Rik; d = 4930455F23Rik.

CCNB1IP1 is a coiled-coil RING domain-containing protein shown to have E3 Ubiquitin ligase activity [23]. The RING domain is characteristic of the E3-ligase family of proteins. To identify potential ubiquitylation targets of CCNB1IP1 and other interacting proteins that might illuminate the molecular mechanism by which this protein participates in crossing over, we conducted a yeast two-hybrid (Y2H) screen of a testis library (Figure 2). Full length CCNB1IP1 was found to be auto-activating under the selective growth conditions of the screen. Progressive *C*-terminal truncations narrowed the autoactivating region to that containing 2 putative Cyclin/Cdk target motifs [5], so these were deleted from the bait vector. Thirty-five interactors were isolated and validated (Table 1; see Methods). None of the CCNB1IP1 interacting genes are known to be essential for meiosis, although *Ggn* (Gametogenetin) has been implicated to have functions beginning in late pachytene spermatocytes [24]. Additionally, the NCBI GEO Profiles database reveals that several of the genes are transcriptionally up-regulated in the testis, with postnatal testis expression increasing in age and peaking during pachytene of meiosis I (accessions GDS3142, GDS605, GDS401). Interestingly, *Hook1* is expressed primarily postmeiotically and is required for proper formation of the sperm head [25], suggesting a possible role for CCNB1IP1 in processes other than recombination.

In light of the Western blot data indicating that *Ccnb1ip1^mei4^*^/*mei4*^ testes produced a deleted version of CCNB1IP1, we hypothesized that the mutant protein might have defective interactions with some subset of the Y2H binding partners, thus potentially explaining the recombination phenotype. We therefore tested the full set of 35 CCNB1IP1 interactors against the mutant allele as bait (signified as *mei4∆ct)*. The *Ccnb1ip1^mei4^* deletion did not ablate interaction with any of the prey clones, however it did reproducibly lead to a “kinetically” weaker interaction as assessed by colony size under stringent growth selection conditions (Figure 2c). If this is reflective of the *in vivo* situation in mice, it is possible that the weaker interactions, coupled with the decreased level of mutant protein, contributes to the phenotype.

Conspicuous amongst the Y2H interactors was SUMO2. This prompted us to consider a potential role for CCNB1IP1 in the SUMOylation cascade. Inspection of the other CCNB1IP1 interacting proteins revealed one notable common motif: Ψ-K-X-E/D (where Ψ is a hydrophobic amino acid; Table 1). This Ψ-K-X-E/D motif is enriched in targets of SUMOylation, and lysine (K) is the residue targeted for SUMO modification in those proteins [26,27]. Notably, of the 13 proteins with kinetic defects in interaction affinity for *mei4∆ct*, 7 (54%) carry the SUMOylation motif with 5/7 possessing the most common form Y/L-K-X-E. The predicted SUMO-proteome has been estimated to be 38% of all similarly analyzed peptides [28].

**Table 1:** Proteins Identified in Two Hybrid Screen.

Kinetically Normal with *mei4*Δ*ct*	Kinetically Defective with *mei4*Δ*ct*
**SUMO2**	4930455F23RIK (3)
AKAP9	YPEL2
SPINK10	1700006A11RIK
1700019N19RIK (2)	EP400
POLR2B	POMP (4)
ENAH	MSL1
H3F3B (3)	DDC8 (2)
5730469M10RIK	HOOK1
PHF12	GGN (4)
MRRF	FHL5
OCIAD1 (2)	4930503B20RIK (2)
B9D1	1700021F07RIK (3)
MORN2	SPATA3 (3)
ATOH8	
SRGN	
PENK1	
BRP44 (3)	
INSL3 (2)	
EMX1	
GSG1 (3)	
OAZ3 (3)	
MIIP	

Note: Proteins with predicted SUMOylation sites are underlined. All clones for the corresponding proteins were isolated with CCNB1IP1Δ*ct* as bait. If multiple, independent clones were obtained for a prey protein, the number is given in parentheses. One of each prey was individually tested for interaction with the MEI4 deletion version. They were subdivided into two kinetic interaction classes as indicated by the two columns.

### 2.3. Motif Analysis of CCNB1IP1 Implicates It Is a SUMO E3 Ligase

**Figure 3:**
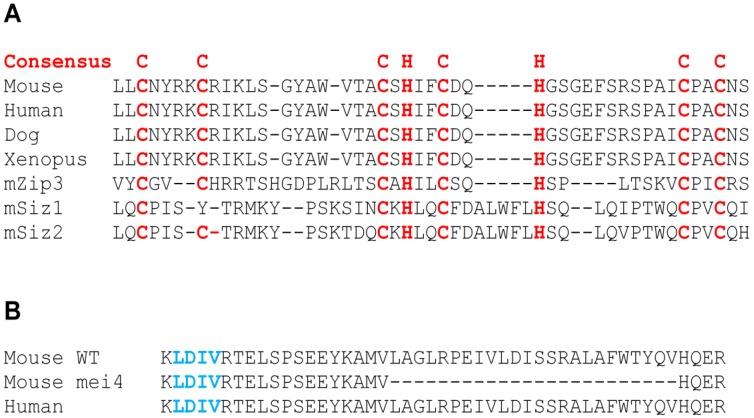
SUMO E3 ligase-like domain conservation in CCNB1IP1. (**A**) Alignments of C3H2C3-type RING domains in four CCNB1IP1 orthologs and other known SUMO E3 ligases; (**B**) CCNB1IP1 contains a canonical SIM motif (blue) just upstream of the region deleted in the *Ccnb1ip1^mei4^* allele.

PIAS4 and other SUMO E3 ligases have been found to interact with SUMO in Y2H assays [29,30,31]. Given that CCNB1IP1 interacts with SUMO2 and other proteins containing consensus SUMOylation sites, we hypothesize that CCNB1IP1 has SUMO E3 ligase function in addition to its reported E3 Ub ligase activity. SUMO E3 ligases often contain a C3H2C3 type RING domain believed to confer interaction specificity to the singular known E2 ligase, UBC9 [32,33]. Alignment of CCNB1IP1 from mouse and other species as well as known SUMO E3 ligases (ZIP3, SIZ1, SIZ2) reveals the presence and conservation of a C3H2C3 type RING domain. Additionally, non-covalent SUMO interacting motifs (SIMs) have been identified within most SUMO E3 ligases [33,34,35]. SIMs are characterized by Ψ-X-Ψ-Ψ where Ψ is V/I or another large hydrophobic residue [34,36]. CCNB1IP1 indeed has such a sequence conserved across mammals (Figure 3b; non-human mammals not shown). 

This putative SIM is just 15 amino acids *N*-terminal to the deletion in the *Ccnb1ip1^mei4^* allele (Figure 3b). Recently, studies of the Pc2 SUMO E3 ligase showed that pairs of charged and hydrophobic amino acids adjacent to the consensus SIM facilitate E3 function [28]. These residues have been proposed to facilitate interactions with SUMO-conjugated UBC9 [36]. The deletion immediately *C*-terminal to the putative CCNB1IP1 SIM, if it contains similar facilitating residues, may impair interaction with UBC9-SUMO *in vivo*.

## 3. Experimental Section

### 3.1. Recombinant Expression of CCNB1IP1 and Anti-CCNB1IP1 Production

cDNA corresponding to amino acids 1–245 of CCNB1IP1 was subcloned into expression plasmid pQE-30 (Qiagen), so as to add a 6X HIS tag. Bacterially expressed peptide was solubilized using standard procedures with the addition of 5 M Urea. The HIS-tagged peptide was purified on a HisPur cobalt resin column according to manufacturer’s protocols (Pierce). The peptide purification was verified via SDS-PAGE and concentrated on a Vivaspin 15R column (SartoriusStedim). The purified CCNB1IP1 was used as immunogen for polyclonal antibody production in rabbit, followed by affinity purification over immobilized CCNB1IP1 as per manufacturer’s procedures (GenScript). Specificity of the IgG was assessed by dot-blot down to 250 pg of recombinant CCNB1IP1.

### 3.2. SDS-PAGE and Western Blotting

SDS-PAGE and Western blotting were performed by standard procedures. Tissues from mice were dounce homogenized in cold lysis buffer (1% SDS, 10 mM EDTA, 50 mM Tris, pH 8.0) supplemented with protease inhibitors (complete^®^, Roche) followed by sonication at 1 s intervals for 30 s. Lysates were boiled for 5 min followed by clearing via centrifugation. Protein concentrations of cleared lysates were measured by the BCA protein assay (Thermo). SDS-PAGE gels were loaded with 25 μg of total protein lysates per lane. Loading controls were performed with anti-β-actin (Sigma A1978) following SDS/2-MeOH stripping of the PVDF membrane. CCNB1IP1 signal was detected with 1:250 dilution of anti-CCNB1IP1 incubated 3 h at 4 °C followed by an hour incubation using anti-rabbit HRP-conjugated antibody. Detection of signals was performed with Pierce^®^ ECL chemiluminescence substrate.

### 3.3. Yeast Two-Hybrid Screen for CCNB1IP1 Interactors

Full length CCNB1IP1 was found to be auto-activating under the Y2H conditions used. Following analysis of various truncations for loss of auto-activation under screen conditions, cDNA of a *C*-terminal truncation of mouse *Ccnb1ip1* corresponding to amino acids 1–245 (encoding what we call CCNB1IP1∆*ct*) was expressed as a “bait” fusion protein with the GAL4 DNA-binding domain. This was constructed in plasmid pGBK. A mouse testis cDNA library in pACT2 (Clontech) was used as “prey” for a protein-protein interaction screen with CCNB1IP1∆*ct*. Interactions with CCNB1IP1∆*ct* were selected by colony growth in the absence of histidine on plates supplemented with 7.5 mM 3-AT. Strongly growing colonies were confirmed under more stringent interaction selection on plates lacking histidine and adenine. pACT2 cDNA clones were isolated and analyzed by DNA sequencing. The CCNB1IP1∆*ct* interactor clones were then directly tested for interaction affinity with the same *C*-terminal truncation from *Ccnb1ip1^mei4^* cDNA (called *mei4*∆*ct*) under the more stringent conditions (Figure 2a). Those clones showing decreased affinity of interaction with *mei4*∆*ct* were confirmed in three independent experiments.

## 4. Conclusions

CCNB1IP1 is required for crossing-over, but there is no known mechanistic linkage to recombination. We conducted the Y2H screen in the hope of identifying proteins of known function that might provide such mechanistic linkage. Although none of the identified interacting proteins have reported roles in crossing-over, our analyses of the aggregate Y2H data suggest a function for CCNB1IP1 as an E3 ligase in the SUMO modification pathway, in addition to the ubiquitin ligase role previously reported in somatic cells [23]. To summarize, we found that CCNB1IP1 interacts with both SUMO2 and proteins containing the consensus SUMOylation motif, properties consistent with computational studies of SUMO E3 ligases [28,31,37]. Furthermore, we observed that CCNB1IP1 contains a C3H2C3 type RING domain, conserved in proven SUMO E3 ligases, that constitutes the interaction surface with the SUMO E2 conjugating enzyme, UBC9. Finally, CCNB1IP1 contains a consensus SIM domain found in the majority of characterized E3s. This sequence has been proposed to function in aiding E3 interaction with SUMO-conjugated UBC9 (not unconjugated UBC9) [38,39]. Whether the putative SIM in CCNB1IP1 actually functions in this manner awaits experimental validation.

There is increasing evidence indicating multiple roles for SUMO modification in regulating DNA repair and meiosis. The *S. cerevisiae* SUMO E3 ligase Zip3 ensures that SC formation is dependent on recombination initiation, and it interacts with a number of recombination proteins including Mre11, Rad51, Msh4 and Msh5 [33,40,41]. The Zip3 ortholog in *C. elegans* is required for meiotic crossover formation and is localized to sites of crossing-over in late Prophase I [42]. Variants in the human ortholog *RNF212* have been associated with influencing genome-wide meiotic recombination rates [43,44]. Furthermore, the synaptonemal complex component protein Zip1 and axial-element protein Red1 have been demonstrated to bind SUMO-conjugated proteins, the latter of which promotes interhomolog exchange [33,45]. Finally, the SUMO pathway is involved in regulating ubiquitylation in DNA damage responses in mammalian cells [9,46].

These results, together with the growing understanding of SUMOylation in higher order eukaryotes, are beginning to shed light on the role for SUMO in DNA damage responses and recombination in meiosis. The defect of *Ccnb1ip1^mei4^* in meiotic crossing over and its putative role as a SUMO E3 ligase offer us a novel element in our understanding of the mechanisms regulating crossover formation. The result of the Y2H screen, which identified a number of putative SUMOylation target proteins with no known roles in meiosis, suggest that further study of CCNB1IP1 will reveal novel mechanisms of meiotic recombination in mammals.
